# A Comb-Shaped Flexible Microelectrode Array for Simultaneous Multi-Scale Cortical Recording

**DOI:** 10.3390/mi17030301

**Published:** 2026-02-28

**Authors:** Suyi Zhang, Jin Shan, Shiya Lv, Yu Liu, Jian Miao, Ziyu Liu, Ezhu Ning, Zhaojie Xu, Juntao Liu, Mixia Wang, Hongyan Jin, Xinxia Cai, Yilin Song

**Affiliations:** 1 State Key Laboratory of Transducer Technology, Aerospace Information Research Institute, Chinese Academy of Sciences, Beijing 100190, China; zhangsuyi23@mails.ucas.ac.cn (S.Z.); danjin16@mails.ucas.ac.cn (J.S.); lvshiya21@mails.ucas.ac.cn (S.L.); liuyu222@mails.ucas.ac.cn (Y.L.); miaojian23@mails.ucas.ac.cn (J.M.); liuziyu242@mails.ucas.ac.cn (Z.L.); ningezhu24@mails.ucas.ac.cn (E.N.); xuzj@aircas.ac.cn (Z.X.); liujuntao@mail.ie.ac.cn (J.L.); wangmixia@mail.ie.ac.cn (M.W.); 2 University of Chinese Academy of Sciences, Beijing 100049, China; 3 Peking University First Hospital, Beijing 100034, China

**Keywords:** flexible micro-electrode array, brain–computer interface (BCI), multi-scale neural recording, cortical microcircuit, audiovisual cross-modal stimulation

## Abstract

High-resolution, multi-modal neural interfaces are essential for advancing systems neuroscience and brain–computer interface technologies. This study designed and fabricated a 128-channel comb-shaped flexible micro-electrode array. The device integrates a biocompatible Parylene substrate with a flexible thin-film microprobe array, enabling simultaneous recording of electrocorticography (ECoG), intracortical local field potentials (LFP), and neuronal action potentials (spikes) from the cortical surface and superficial layers. Microelectrode sites were modified with platinum black nanoparticles, significantly reducing impedance. In vivo experiments in rats demonstrated the array’s ability to capture high-fidelity signals across different recording depths. Key findings included the acquisition of opposing LFP trends and polarity reversals between adjacent channels, reflecting local microcircuit dynamics. The array also reliably recorded neural activity during audiovisual cross-modal sensory stimulation. These results validate the device as an effective tool for multi-scale electrophysiology, successfully balancing high spatial resolution and signal quality with minimal tissue invasiveness, thereby offering significant potential for fundamental research and neural engineering applications.

## 1. Introduction

The precise recording of neural activity forms the cornerstone for advancing fundamental brain science, neural engineering, and brain–computer interface (BCI) technologies. The cortical region, a critical structure for processing sensory input, motor commands, and higher-order cognitive functions in the brain, holds a wealth of valuable electrophysiological information within its surface and superficial layers. This includes signals such as electrocorticography (ECoG) obtainable from within or beneath the dura mater, as well as local field potentials (LFPs) and neuronal action potentials (spike) that can be captured intracortically [1]. Therefore, developing micro-electrode technology capable of high spatiotemporal resolution recording across both the cortical surface and intracortical regions is crucial for unraveling neural coding mechanisms, establishing stable neural interfaces, and advancing clinical neuromodulation methodologies.

However, traditional scalp electroencephalography (EEG) suffers from low signal amplitude and poor spatial resolution due to the skull’s filtering effect, making it inadequate for capturing fine-grained neural information [2]. In contrast, intracranial electrocorticography (ECoG) and intracortical microelectrodes can provide significantly higher signal-to-noise ratios and superior spatial localization, establishing them as essential tools in modern neuroscience research and clinical monitoring [3]. Currently widely used silicon-based rigid implantable electrode arrays, such as the Utah array, are capable of penetrating directly into the cortical tissue to record single-unit or sparse multi-unit spike signals, offering millisecond-level temporal resolution and excellent spike detection capability [4]. However, due to their significantly higher rigidity compared to brain tissue, these rigid electrodes inevitably generate substantial relative micromotion under the influence of physiological brain pulsations. This leads to chronic inflammation [5] and glial scar formation [6] in the surrounding neural tissue, resulting in the long-term degradation of signal quality. Furthermore, the channel density of such arrays is inherently limited by the fabrication constraints of silicon-based processes [7], posing a significant challenge for further scaling. While these high-invasiveness, high-resolution devices have played a crucial role in fundamental neuroscience research, their performance in terms of long-term implantation stability, biocompatibility, and cross-species applicability [8] requires substantial improvement.

To overcome the issues of high invasiveness and poor long-term stability associated with rigid electrodes, flexible cortical surface electrodes have garnered significant attention in recent years [9]. Flexible materials such as Parylene [10] and polyimide (PI) [11] possess a low Young’s modulus, excellent biocompatibility, and controllable thickness at the micrometer scale. These properties enable the electrodes to conform tightly to the cortical surface [12,13,14], significantly reducing contact impedance and enhancing signal quality [15]. Such flexible surface electrodes enable the implementation of high-density, large-area array configurations, demonstrating excellent performance in applications such as sensory function mapping and epileptic focus localization [16]. However, conventional flexible cortical electrodes are predominantly limited to signals acquired at the brain surface. Due to the distance between the electrode and neurons, their capability to record spike signals is inherently limited [17], making it challenging to concurrently capture both large-scale neural field activity and single-unit spiking activity. Meanwhile, Parylene-based flexible probes have demonstrated reduced glial scarring and improved long-term recording stability in both acute and chronic settings [18,19]. Recent advances in mechanical modeling have further optimized probe geometry to minimize insertion trauma [20]. Despite these advances, most flexible depth probes remain dedicated solely to intracortical recording and lack the capability to simultaneously acquire surface ECoG signals from the same device [21].

Concurrent recording of ECoG and intracortical spikes has been recognized as a powerful strategy for linking mesoscale network dynamics with single-neuron resolution [22]. Previous efforts have addressed this need by implanting separate devices—e.g., combining a surface ECoG grid with one or more depth probes—or by integrating electrodes on separate shanks of a single rigid carrier [23]. However, these approaches often involve complex assembly procedures, increased tissue footprint, or limited spatial alignment between recording modalities. To date, achieving hybrid surface and depth recording using a single monolithic flexible microelectrode array (MEA) with unified substrate and homogeneous microfabrication process remains an underexplored engineering challenge [24].

In this study, we present a 128-channel comb-shaped flexible cortical micro-electrode array that integrates a fully flexible Parylene substrate with a set of thin-film microprobes distributed across a single device. The key innovation lies not merely in the use of flexible polymers, but in the heterogeneous configuration of the array: sixteen probes are arranged such that some can be conformally placed on the cortical surface while adjacent probes are inserted into superficial cortical layers. This unique layout enables, for the first time, the simultaneous recording of ECoG, LFP, and spike signals from both surface and intracortical domains using a single monolithic MEA. The device thus bridges a critical technological gap between conventional flexible surface electrodes—which lack access to unit activity—and rigid penetrating probes—which often incur tissue damage and signal degradation over time. Recent developments in stiffness-tunable probes [25] and magnetically actuated dynamic electrodes [26] represent complementary strategies for minimally invasive implantation, while our work provides a distinct solution based on heterogeneous monolithic integration.

Against this backdrop, the 128-channel comb-shaped flexible Parylene cortical micro-electrode array designed and fabricated in this work presents significant innovations. It utilizes a Parylene substrate to provide excellent mechanical compliance and biostability. The unique comb-shaped structure enables shallow intracortical penetration for spike signal acquisition, while its large conformal contact area ensures high-quality ECoG recording. Furthermore, this electrode array achieves a high-density layout with up to 128 channels, markedly enhancing spatial sampling capability. This makes it suitable for multi-sensory (e.g., visual, auditory) stimulation experiments, functional cortical mapping, and investigations into neural network dynamics. The stability and multi-level signal acquisition capability of this array have been successfully validated in rat auditory–visual stimulation experiments, demonstrating its considerable potential for both fundamental neuroscience research and implantable brain–computer interface technologies. High-density µECoG arrays have recently demonstrated utility in tracking cortical dynamics in awake animals [27], further supporting the translational relevance of our approach.

## 2. Materials and Methods

### 2.1. Reagents and Instruments

Phosphate-buffered saline (PBS, 0.1 M, pH 7.4) was purchased from Sigma-Aldrich (St. Louis, MO, USA). Physiological saline (0.9% sodium chloride) was obtained from Shuanghe Corporation (Beijing, China). The Chloroplatinic acid (H_2_PtCl_6_) and lead acetate [(CH_3_COO)_2_Pb] were supplied by Sinopharm Chemical Reagent (Shanghai, China). Isoflurane and a small animal anesthesia machine were purchased from RWD Life Science Co., Ltd. (Shenzhen, China).

The modification of the flexible cortical micro-electrode array was performed using an electrochemical workstation (Gamry Reference 600, Gamry Instruments, Warminster, PA, USA). Electrophysiological signals were acquired by a custom-built 128-channel neural signal acquisition system which is independently developed by Aerospace Information Research Institute, Chinese Academy of Sciences (Beijing, China).

### 2.2. Electrode Design and Fabrication

#### 2.2.1. Design of the Flexible Cortical Electrode Array

**Figure 1:**
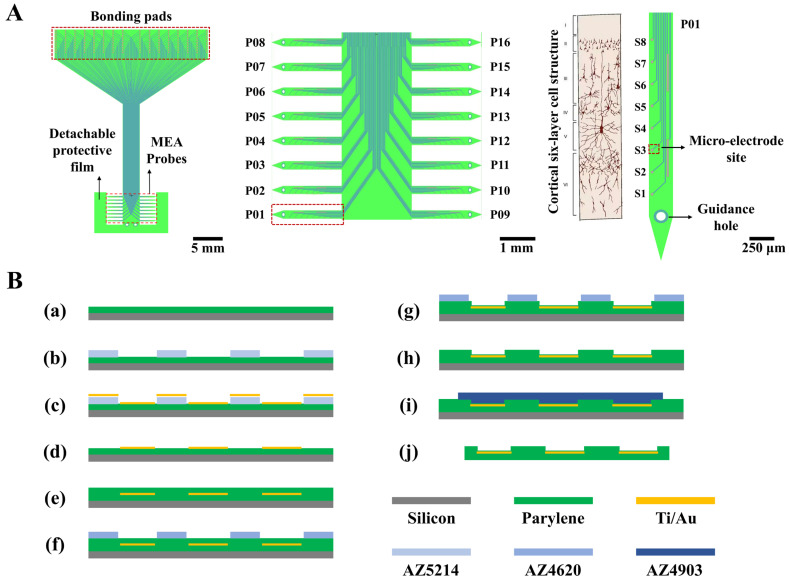
Structural design and fabrication process flow of the flexible cortical electrode array. (**A**) Schematic illustration of the novel flexible cortical electrode array design, including the overall profile (left), the sixteen individual probes (middle), and a single probe aligned with the six-layer cortical cellular architecture (right). (**B**) Flowchart of the fabrication process for the flexible electrode array. Steps include: (**a**) deposition of the Parylene substrate layer on a silicon wafer; (**b**–**d**) patterning of the metal layer; (**e**–**h**) deposition and patterning of the insulation layer to open vias; (**i**,**j**) release of the electrode from the silicon carrier substrate.

The structure of the designed 128-channel, comb-shaped flexible cortical electrode array is illustrated in Figure 1A. The entire device comprises sixteen individual thin-film MEA probes (P01~P16), whose tips are temporarily anchored to a common detachable protective film to prevent the slender probes from deformation during preparation. Each probe is equipped with eight electrophysiological recording microelectrode sites (S1~S8). When conformally attached to the cortical surface, the probes splay outward, enabling coverage of a broader area. This design facilitates the investigation of information transfer across cortical regions and allows for the recording of intracranial electrocorticography (ECoG) signals over an extended field. The eight sites are also designed to span the six layers of the cortical column upon insertion. Each probe features a guidance hole at its tip, permitting sequential implantation into deeper cortical layers using a corresponding mold. This capability enables the recording of more precise local field potential (LFP) and action potential (spike) signals within the cortex. The central interconnect region mimics the substrate of a Utah array, designed to float freely with the brain surface, thereby mitigating tissue damage caused by mechanical mismatch.

The key geometrical parameters are as follows: each probe has a total length of 3 mm and a width of 250 μm, with an inter-probe pitch of 500 μm. The 128 recording sites have a diameter of 30 μm, and the spacing between microelectrode sites on the same probe is 250 μm. The interconnects are 8 μm wide with a spacing of 10 μm. The reference electrode measures 420 μm in length and 20 μm in width, with a 10 μm wide interconnect. The bonding pads measure 200 × 300 μm with a center-to-center spacing of 225 μm. The guidance hole has a diameter of 100 μm.

This electrode design can cover an area of approximately 1 cm × 1 cm over the rat cerebral cortex. It is capable of simultaneously monitoring multiple brain regions, including the left and right primary (M1) and secondary (M2) motor cortices, the primary (V1) and secondary (V2) visual cortices, and the primary somatosensory cortex (S1). Furthermore, the probe length is sufficient to access all six cortical layers, making it possible to study cross-layer information processing within the cortical column.

#### 2.2.2. Preparation of the Substrate Layer

A clean 4-inch silicon wafer served as the carrier substrate. After treatment with oxygen plasma (150 W, 180 s), it was placed into a Parylene deposition system. An 8 μm thick flexible Parylene substrate layer was formed by the chemical vapor deposition of 26 g of Parylene-F over an 8 h period (Figure 1B(a)).

#### 2.2.3. Patterning of the Metal Layer

The sample was treated with oxygen plasma (50 W, 90 s) and then baked on a hotplate at 120 °C for 120 s to remove surface moisture. Positive photoresist AZ5214 was spin-coated onto the flexible substrate at 1600 rpm for 60 s. This was followed by a soft bake on a 100 °C hotplate for 150 s; the temperature was strictly controlled at 100 °C to prevent photoresist cracking. After complete cooling, mask alignment and exposure were performed for 1 s. Subsequently, a reversal bake at 100 °C for 210 s and a flood exposure for 12 s were conducted to convert the AZ5214 photoresist from positive to negative tone, ensuring the exposed regions would remain after development while the unexposed areas would be dissolved. After photolithography, the photoresist was developed in a 0.6% sodium hydroxide solution (Figure 1B(b)). A hard bake was then performed in an 80 °C oven for 20 min. A metal layer of 300 Å Ti and 2000 Å Au was deposited onto the sample via evaporation deposition (Figure 1B(c)). The deposition was performed at a base pressure of 2 × 10^−6^ Torr, with an electron gun power of 36 kV and a deposition rate of 5 Å/s. Finally, the sample was immersed in acetone. The photoresist mask, which defined trenches for the electrode sites, interconnects, and bonding pads, was dissolved. The metal deposited elsewhere was consequently lifted off, leaving behind the desired electrode patterns. Any residual photoresist was completely removed by oxygen plasma treatment (150 W, 180 s) (Figure 1B(d)).

#### 2.2.4. Deposition and Patterning of the Insulation Layer

The sample was placed into the Parylene deposition system again. A total of 19.5 g of Parylene-F was evaporated over 6 h to form a 6 μm thick insulation layer (Figure 1B(e)); this thickness was chosen to prevent layer fracture. Positive photoresist AZ4620 was spin-coated at 1600 rpm for 60 s and cured in an 80 °C oven for 40 min, forming a 10 μm thick photoresist film. The temperature was strictly maintained at 80 °C, which is optimal for forming crack-free photoresist films. After complete cooling, mask alignment and exposure were performed for 10 s to define the electrode sites and bonding pads. After development in 0.6% sodium hydroxide, the sample underwent a post-exposure bake at 80 °C for 30 min. The electrode sites and bonding pads were exposed, while the interconnects remained protected by the photoresist (Figure 1B(f)). The Parylene insulation layer over the sites and pads was then etched away using oxygen plasma etching (600 W, 8 min) to open vias (Figure 1B(g)). Since oxygen plasma etches both Parylene and the positive photoresist, it was crucial to ensure the photoresist mask was sufficiently thick to protect the Parylene covering the interconnects. Finally, the sample was immersed in acetone to remove the remaining photoresist (Figure 1B(h)).

#### 2.2.5. Release of the Electrode from the Silicon Carrier

The sample was cleaned with oxygen plasma (100 W, 30 s) to remove chemical deposits from the site and pad surfaces. Positive photoresist AZ4903 was then spin-coated at 2500 rpm for 60 s and cured in an 80 °C oven for 50 min, forming a 12 μm thick film. After complete cooling, mask alignment and exposure of the non-electrode areas were performed for 60 s. Following development in 0.6% sodium hydroxide (Figure 1B(i)) and a post-bake at 80 °C for 40 min, the electrode patterns were protected by the photoresist mask. The exposed surrounding areas were etched away using oxygen plasma (600 W, 12 min), leaving only the desired electrode structures. The sample was immersed in acetone to remove the photoresist mask and release the electrode array. Subsequently, it was immersed in ethanol to rinse away residual acetone and other chemicals, completing the release of the flexible electrode array (Figure 1B(j)).

#### 2.2.6. Bonding and Packaging

Individual electrodes were released for the flip-chip bonding process. Tin solder balls with a diameter of 200 μm were placed on a custom-designed flexible printed circuit board (FPCB). The FPCB was placed on a hotplate to melt the solder balls. Under a microscope, the electrode bonding pads were precisely aligned with the molten solder balls on the FPCB, and the assembly was returned to the hotplate to form the connections. After flip-chip bonding, pin headers were soldered to the FPCB. Thus, the signals detected by the electrode could be routed via the FPCB and pin headers to the external signal acquisition instrument. Finally, the assembly was encapsulated with a protective layer of room-temperature-vulcanizing (RTV) silicone.

### 2.3. Modification of the Flexible Cortical Electrode Array

To reduce the electrode impedance, platinum black nanoparticles (PtNPs) were electrodeposited onto the electrode sites, leveraging their high surface-area-to-volume ratio advantage [28]. This nano-modification technique creates a sensitive sensing interface, enabling the electrodes to achieve lower impedance and an increased effective contact area with cortical cells. The electrodeposition of PtNPs was performed using a standard three-electrode system, comprising the electrode site as the working electrode, an Ag/AgCl reference electrode, and a platinum counter electrode. Amperometric deposition was conducted at a constant potential of −0.8 V for 50 s. The plating solution was a mixture of 24 mM H_2_PtCl_6_ and 2.1 mM Pb(CH_3_COO)_2_. During the deposition process, sonication was applied to promote uniform nanoparticle growth.

### 2.4. Animal Experimental Procedures

Male Sprague-Dawley (SD) rats weighing 250 g were used in this study. All rats were purchased from Beijing Vital River Laboratory Animal Technology Co., Ltd. (Beijing, China). The animals were individually housed under standard conditions with a 12 h light/dark cycle. The ambient temperature was maintained at 25 ± 3 °C, and the humidity was controlled between 50% and 70%. Food and water were provided ad libitum. All animal experiments were approved by the Beijing Laboratory Animal Welfare and Ethics Committee (Beijing, China) and were conducted in accordance with the guidelines of the Institutional Animal Care and Use Committee (IACUC) of the Aerospace Information Research Institute, Chinese Academy of Sciences (AIRCAS, Beijing, China).

**Figure 2:**
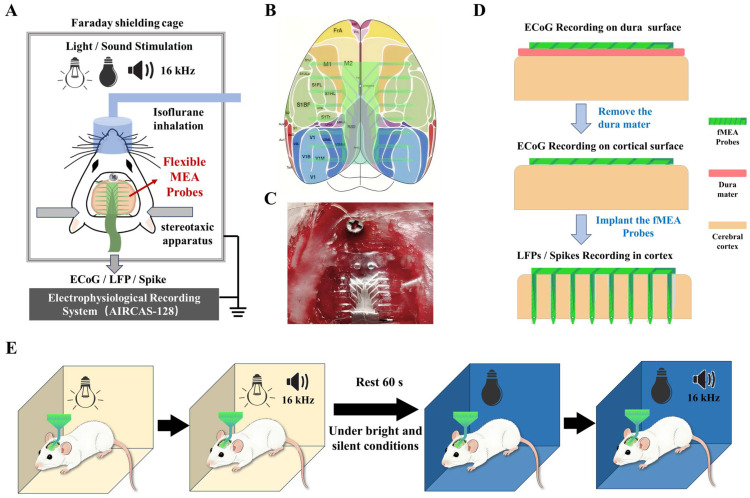
Schematic diagram of the experimental procedures. (**A**) Animal experiment overall plan schematic diagram. (**B**) Schematic illustration of the placement of the flexible cortical electrode array on the cortex. Different probes cover areas including the primary (M1) and secondary (M2) motor cortices, the primary (V1) and secondary (V2) visual cortices, and the primary somatosensory cortex (S1). (**C**) Illustration of the electrode recording configurations: on the dura mater surface, beneath the dura mater on the pial surface, and implanted within the cortical tissue. (**D**) Photograph of the actual surgical procedure showing the flexible cortical electrode array attached to the cortical surface of an anesthetized rat. (**E**) Diagram of the animal experimental paradigm, consisting of four sequential conditions: bright light, bright light with an additional 16 kHz tone, darkness, and darkness with an additional 16 kHz tone.

During the surgical procedure, the rat was anesthetized using an isoflurane anesthesia machine and securely fixed in a stereotaxic frame. A dental drill was used to create a 2 × 2 cm cranial window. The coordinates for the window were defined relative to the bregma: 1 cm lateral on each side, 0.5 cm anterior, and 1.5 cm posterior. A skull screw was fixed above the bregma to serve as the ground reference. Subsequently, the bone flap within the window was carefully removed. Postoperatively, the rat was placed in a shielded cage to eliminate external electro-magnetic interference (Figure 2A)

A schematic diagram of the targeted brain regions for electrode recording is shown in Figure 2B. As shown in Figure 2C, a photograph of the actual surgical procedure is presented.

The flexible micro-electrode array was then positioned in three configurations: on the surface of the exposed dura mater, beneath the dura mater on the pial surface, and with the probes implanted intracortically (Figure 2D). All three recording configurations were performed sequentially on the same animal and the same electrode array during a single acute experiment. Each configuration lasted approximately 20 min, including the sensory stimulation protocol and rest periods.

To achieve precise and minimally traumatic implantation, we developed a custom manual implantation method using an electrochemically etched and sterilized tungsten wire with a tapered tip and a flat shoulder. The tip diameter of the tungsten wire is less than 100 μm, allowing it to easily pass through the 100 μm guidance hole, while the shoulder diameter matches the original wire diameter (500 μm) and acts as a mechanical stop, enabling reliable pickup of the probe tip.

For each probe designated for intracortical insertion, the tungsten wire is carefully threaded through the guidance hole until the shoulder engages, allowing the probe tip to be gently lifted and the slender probe tip to be folded upward. The wire is then held vertically by hand, and the probe is inserted perpendicularly into the cortical tissue at a steady speed until the shoulder contacts the predetermined intracortical cellular layer. This approach ensures that the flexible microneedles penetrate the pia mater and enter the superficial cortical layers without buckling or excessive dimpling.

After insertion, the tungsten wire is disengaged by retracting it horizontally, leaving the probe tip securely embedded in the cortex while the central floating interconnect region remains on the cortical surface. This method allows sequential, high-precision implantation of multiple probes on the same monolithic array with minimal tissue damage.

To provide sensory stimulation, environmental light and sound were modulated. The recording protocol consisted of four distinct conditions, each lasting 60 s: (1) bright white light, (2) bright white light combined with a 16 kHz tone, (3) darkness, and (4) darkness combined with a 16 kHz tone (Figure 2E). A 60 s rest period was introduced before the dark conditions to minimize potential effects from prior auditory stimulation. The 16 kHz pure tone was repeated every 3 s throughout each 60 s stimulation window, with each tone burst lasting 1 s. The sound pressure level was approximately 70 dB SPL at the animal’s ear. This four-condition stimulation cycle was repeated three times for each recording configuration, yielding a total recording time of approximately 60 min per configuration.

A total of three independent acute experiments were conducted using three separate SD rats and three individual electrode arrays. All three configurations (dura surface, subdural pial surface, and intracortical implantation) were tested in each animal with the same electrode. Neural signals from all 128 channels were recorded and analyzed. The data presented in this study are representative of consistent findings observed across all three experiments.

Using this in vivo recording technique, the full spectrum of electrophysiological signal changes across the three electrode configurations and four stimulus conditions was successfully captured. Neural signals were acquired using the custom-built 128-channel neural signal acquisition system (AIRCAS-128). EcoG and local field potentials (LFPs) were recorded at a sampling rate of 1 kHz, while action potentials (spikes) were recorded at 30 kHz.

### 2.5. Linear Signal-to-Noise Ratio Calculation

To quantitatively assess and compare the quality of neural signals acquired by the flexible cortical electrode array under different recording configurations, the linear signal-to-noise ratio (SNR) was calculated based on the peak-to-peak voltage (Vpp) and the overall root mean square (RMS) value. Specifically, stable signal segments were extracted from raw signals recorded by each channel under defined experimental conditions. First, the peak-to-peak voltage of each segment was calculated to characterize the effective dynamic range of the neural activity. Subsequently, the root mean square (RMS) value of the entire signal segment was computed, representing the total energy comprising both the neural signal and background noise. The linear signal-to-noise ratio was then derived using the following formula:

SNR_linear = Vpp/(2 × RMS),

This metric provides a quantitative basis for objectively comparing signal quality across different recording depths.

## 3. Results

### 3.1. Characterization and Performance Evaluation of the Flexible Cortical Electrode Array

**Figure 3:**
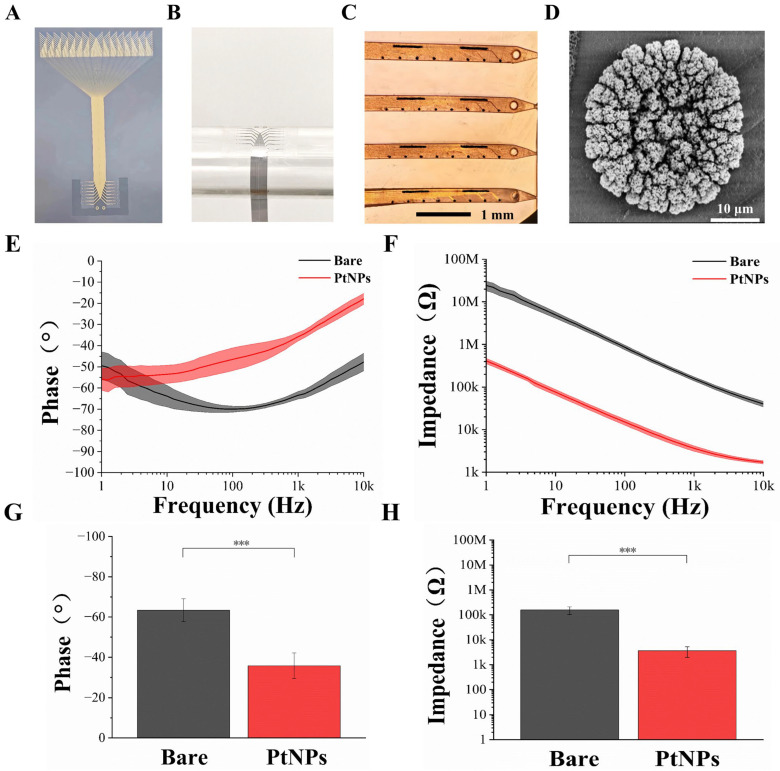
Characterization of the flexible cortical electrode array and performance evaluation after PtNPs modification. (**A**) Optical image of the entire fabricated device. (**B**) Mechanical flexibility test performed by conforming the electrode array to a cylindrical tube (~1 cm diameter). (**C**) Microscopic image of the flexible cortical electrode array after PtNPs modification. (**D**) Scanning electron microscopy (SEM) image of an electrode site modified with PtNPs. (**E**) Phase angle comparison between bare and PtNPs-modified electrode sites across a frequency range of 1 Hz to 10 kHz. (**F**) Impedance magnitude comparison between bare and PtNPs-modified electrode sites across a frequency range of 1 Hz to 10 kHz. (**G**) Comparison of the average phase angle at 1 kHz for bare and PtNPs-modified recording sites (*n* = 10, *** *p* < 0.005). (**H**) Comparison of the average impedance magnitude at 1 kHz for bare and PtNPs-modified recording sites (*n* = 10, *** *p* < 0.005).

The fabricated flexible cortical electrode array is shown in Figure 3A. Its multi-probe design fulfills the requirement for detecting electrophysiological activity from both neuronal populations and single cells within the cortex. To evaluate the mechanical flexibility of the electrode, it was conformed to a cylindrical tube with a diameter of approximately 1 cm (Figure 3B). The electrode demonstrated excellent conformability, perfectly adhering to the curved surface and confirming its superior flexibility. The Young’s modulus of Parylene, the structural material of our device, has been reported to be in the range of 2–5 GPa [29], which further supports the inherent flexibility of the electrode. Through the design, fabrication, and modification processes, the flexible microelectrode array was rendered suitable for in vivo animal experimentation.

Figure 3C presents the surface morphology of an electrode site after modification with electrodeposited platinum nanoparticles (PtNPs). All recording sites were uniformly covered with black particulate matter, indicating successful PtNPs deposition. Scanning electron microscopy (SEM) observation (Figure 3D) revealed a dense layer of PtNPs formed on the site surface, effectively increasing the specific surface area of the electrophysiological recording sites.

Electrode impedance and phase are critical parameters for detecting weak neural signals. To assess the impedance and phase characteristics of the flexible cortical electrode array, electrochemical impedance spectroscopy (EIS) was employed. A standard three-electrode system was used to measure the impedance of a single electrode site (30 μm diameter) in phosphate-buffered saline (PBS, pH 7.4) at room temperature. EIS spectra were recorded from 1 Hz to 10 kHz using a Gamry electrochemical workstation. The average impedance magnitude and phase angle of the electrode sites, before and after modification, are clearly displayed in Figure 3E,F, respectively. A significant reduction in impedance magnitude was observed across the frequency range from 1 Hz to 10 kHz. Concurrently, the phase angle increased, leading to a corresponding decrease in phase delay over the same frequency range. These results indicate that the modified microelectrode array (MEA) sites exhibit lower impedance and higher phase angles. The electrodeposition of PtNPs effectively improved both the impedance and phase characteristics.

Given that the fundamental frequency of action potentials is typically around 1 kHz [30], we focused our characterization on this frequency. As shown in Figure 3H, the average impedance of bare electrode sites was 157.03 ± 53.98 kΩ. Following PtNP modification, impedance dropped drastically to 3.64 ± 1.67 kΩ—a reduction of over 97%. Correspondingly, the phase angle at 1 kHz shifted from −63.41° to −35.81° (Figure 3G), indicating a shift toward a more resistive (less capacitive) interface. This phase angle increase (reduction in absolute value) reflects improved charge transfer kinetics and lower impedance at the electrode–electrolyte interface [31]. A more resistive phase behavior is associated with reduced signal attenuation and phase delay, which are beneficial for high-fidelity recording of fast neural activities such as action potentials [32,33].

### 3.2. Characteristics of Cortical Population Electrophysiological Activity in Rats Under Different Stimulus Conditions

In neuroelectrophysiological research, recording the electrical activity of neuronal populations at and near the cortical surface is pivotal for unraveling the mechanisms of brain function. Based on the spatial relationship between the recording electrode and the cortex, the primary signals can be categorized into two types: Local Field Potentials (LFPs) and Electrocorticography (ECoG).

Local Field Potentials (LFPs) typically refer to recordings of population electrical activity obtained from within the cortical tissue [34]. They primarily reflect the spatial summation of synaptic post-potentials (mainly dendritic) from neurons within a few hundred micrometers of the electrode tip [35]. With high spatial resolution (approximately 0.1–1 mm), LFPs can delineate the fine-grained activity of local microcircuits, serving as a key signal for studying neuronal communication in the brain [36]. Electrocorticography (ECoG), on the other hand, records population electrical activity from the surface of the dura mater or the subdural pial surface [37]. It integrates the synchronized activity of vast neuronal populations across a broader cortical area (several square centimeters) [38]. ECoG signals are characterized by their stability, strong resistance to interference, and spatial resolution intermediate between scalp electroencephalography (EEG) and LFP (approximately 0.5–2 cm) [39].

Together, these two signals constitute a multi-scale observational window, spanning from local neural networks to macroscopic cortical regions. The following section first analyzes these electrical activities originating from neuronal populations.

**Figure 4:**
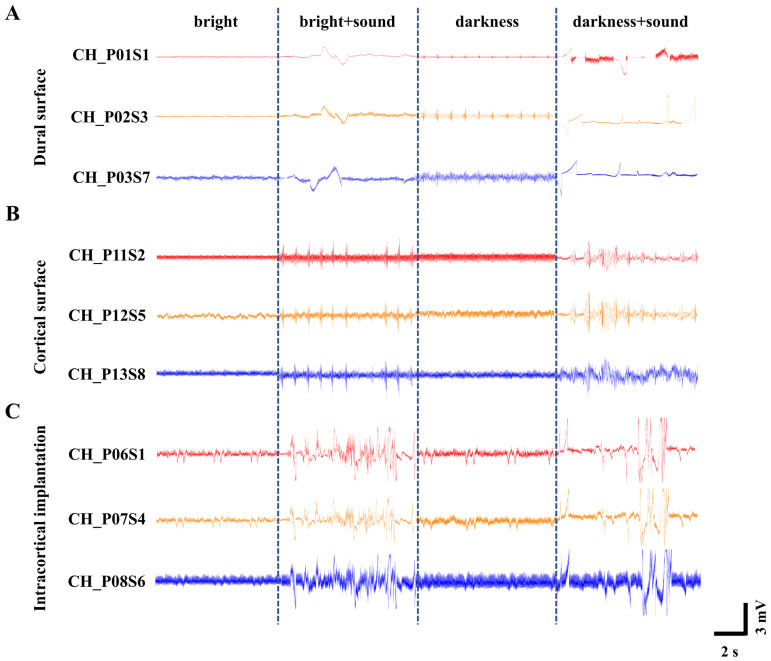
Recordings of neural population activity under different light and sound stimulus conditions. (**A**) ECoG signals recorded on the dura mater surface. (**B**) ECoG signals recorded on the subdural pial surface following dura removal. (**C**) LFP signals recorded with intracortical implantation.

Neural activity of cortical populations was modulated using visual (light/dark) and auditory (16 kHz tone) stimuli. Leveraging the broad spatial coverage of the flexible cortical electrode array, large-scale neural activity dynamics across the rat cortex were captured under three distinct recording configurations: on the dura mater surface (Figure 4A), on the subdural pial surface (Figure 4B), and with intracortical implantation (Figure 4C). Figure 4 shows the real-time population electrical activity waveforms (200 Hz low-pass filtered) recorded by electrode array under three detection methods in representative channels, a phenomenon commonly observed across multiple channels.

To quantitatively compare the signal quality across the three recording configurations, we calculated the linear signal-to-noise ratio (SNR) for each configuration using the formula described in Section 2.5. The analysis yielded average SNR values of 2.98 ± 0.21 for dural surface recording, 4.16 ± 0.35 for subdural pial surface recording, and 8.32 ± 0.54 for intracortical implantation. This clear ascending trend demonstrates that more invasive recording configurations provide progressively higher SNRs, primarily driven by the larger amplitude of neural signals captured closer to the neuronal sources.

We note that the LFP traces shown in Figure 4A (dural surface) appear smoother and visually less noisy than those in Figure 4C (intracortical). This apparent contradiction arises because Figure 4 displays only low-frequency local field potential components (<300 Hz). Surface recordings benefit from the low-pass filtering effect of the dura and cerebrospinal fluid, which attenuates high-frequency noise and makes the LFP traces appear smoother. However, the SNR is a comprehensive metric that accounts for both signal amplitude and noise energy across the entire frequency spectrum. Intracortical recordings offer two distinct advantages that are not visible in the LFP-only display: (1) they capture substantially larger LFP amplitudes due to closer proximity to the neuronal current sources, and (2) they enable recording of high-frequency neural activity including action potentials (spikes), which are completely absent in surface recordings [40].

Furthermore, concurrent surface and depth recordings in human patients have revealed that only 26–34% of spike activity co-occurs between cortical surface and intracortical electrodes, with the majority (65–74%) of neural events captured exclusively by penetrating electrodes [41]. This provides direct clinical evidence that surface recordings miss a substantial portion of high-frequency neural information, and that the superior SNR of intracortical recordings reflects genuine access to a richer neural signal repertoire rather than merely lower noise.

Thus, the numerical SNR values and the visual appearance of the LFP traces are not contradictory; rather, they reflect the fundamental differences in frequency content and signal composition captured by each recording modality. The superior SNR of intracortical recordings validates the advantage of penetrating microelectrode arrays for high-fidelity neural interfacing, a conclusion consistently supported by recent developments in high-density µECoG and depth electrode technologies [42,43].

In contrast, a horizontal comparison across the different light and sound environments revealed distinct waveform patterns. Under normal bright conditions, the waveform amplitude exhibited gentle fluctuations without significant variation. This suggests that with sustained visual input, the cerebral cortex remains in a relatively stable state, where the spontaneous firing of neurons is likely moderately synchronized and at a low level, resulting in a lower baseline (0.5 mV~1 mV).

The 16 kHz tone is among the frequencies to which rat hearing is most sensitive, characterized by one of the lowest auditory thresholds [44]. Therefore, the introduction of a 16 kHz auditory stimulus under bright conditions elicited an increase in waveform amplitude to approximately 2 mV–5 mV. This indicates that a sudden auditory stimulus against a stable visual background likely triggered significant cross-modal sensory integration. Multisensory integration cortices in the brain responded to this unexpected stimulus, leading to an elevation in the amplitude of evoked potentials.

In contrast, when the light was extinguished and the environment became completely dark, the overall waveform baseline rose to about 1 mV~1.5 mV, suggesting heightened brain activity [45]. This phenomenon may be attributed to the absence or reduction in visual input, potentially leading to enhanced spontaneous activity within the cerebral cortex [46], thereby placing it in a heightened state of “preparedness” to cope with uncertainty. Consequently, when the 16 kHz tone was introduced under this dark condition, the waveform fluctuations became more pronounced, with amplitudes reaching approximately 3 mV~6 mV. Against an elevated baseline, the same auditory stimulus may have been perceived as more novel or salient, eliciting larger-amplitude event-related potentials, which reflect more advanced cognitive processing. These experimental observations align fully with established principles of cross-modal sensory enhancement in rats [47].

### 3.3. Spike Firing Patterns of Secondary Visual Cortex (V2) Neurons Under Different Conditions

To investigate the firing characteristics of cortical neurons in rats under different conditions, we further analyzed the patterns of action potentials (spikes). Firing rate (spikes per second) was used as the parameter to assess the intensity of neuronal activity. Spikes were identified, extracted, and isolated from noise using principal component analysis (PCA) and K-means clustering algorithms [48].

**Figure 5:**
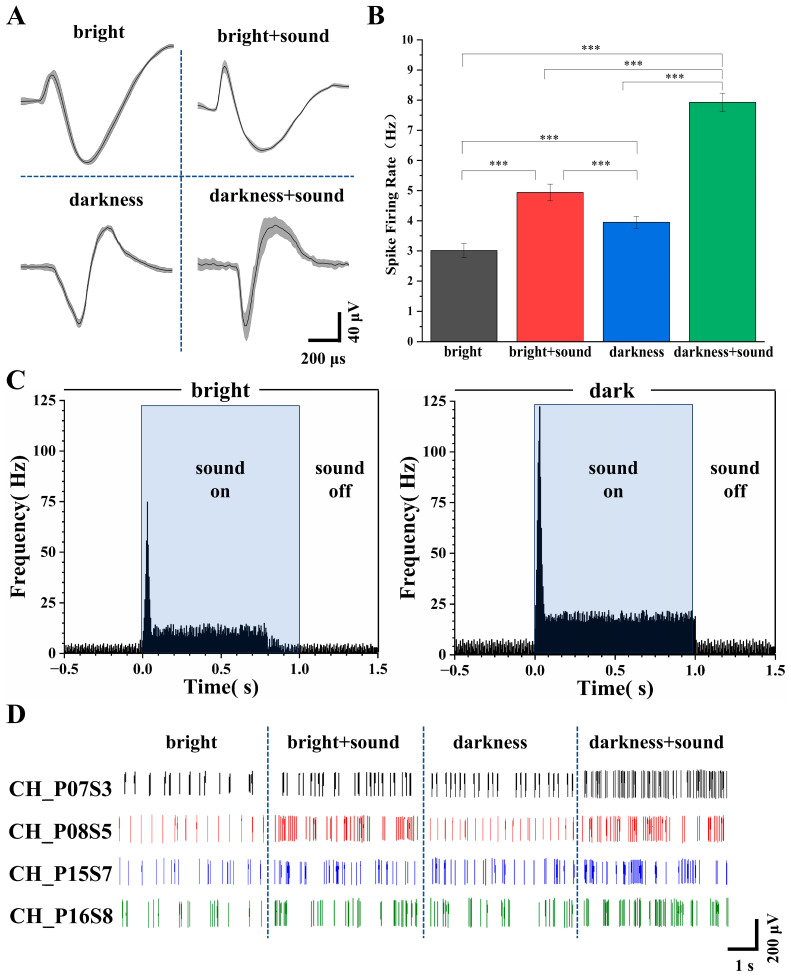
Analysis of V2 neuronal firing under different conditions. (**A**) Average spike waveforms recorded under the four experimental conditions. (**B**) Firing rates under the four conditions (*n* = 30, *** *p* < 0.005). (**C**) Post stimulus time histograms (PSTHs) of V2 neurons in response to auditory stimuli under light (left) and dark (right) conditions. (**D**) Representative real-time signal diagram of spike firing across the different conditions.

Based on the spike sorting results, we analyzed the average spike waveforms of secondary visual cortex (V2) neurons under the four different environmental stimulus conditions. As shown in Figure 5A, the average waveforms remained consistent within the same lighting condition, indicating that neither the ion channels nor the membrane potentials of the rat neurons were altered before and after the introduction of the auditory stimulus. This waveform stability indicates that the baseline functional state of voltage-gated sodium and potassium channels—as well as the resting membrane potential—remained unaltered during the 60 s recording epoch within each lighting condition, providing a stable physiological reference for assessing cross-modal effects.

Following the addition of the sound stimulus, waveform duration increased and peak amplitude became more pronounced (Figure 5A). These waveform changes are direct readouts of underlying ionic conductance modulation: (1) increased duration reflects slowed repolarization due to reduced voltage-gated potassium channel activity (e.g., Kv3.1/Kv1), which regulates action potential width and calcium influx; (2) enhanced peak amplitude indicates increased sodium channel availability or faster activation kinetics, improving depolarization efficiency [49]. Extracellular spike waveform features—particularly trough-to-peak duration and peak amplitude—are well-established markers of neuronal subtype and physiological state, and can be dynamically modulated by sensory inputs [50]. Thus, the observed waveform broadening and peak enhancement under audiovisual stimulation reflect auditory-driven modulation of V2 membrane excitability and ion channel function, consistent with cross-modal physiological plasticity.

Subsequently, the firing rates under the four conditions were calculated. As depicted in Figure 5B, the firing rates of V2 neurons under the four conditions were 2.96 ± 0.28 Hz, 4.97 ± 0.22 Hz, 3.92 ± 0.25 Hz, and 7.96 ± 0.20 Hz, respectively.

To investigate the temporal dynamics underlying the firing rate differences, we constructed post stimulus time histograms (PSTHs) aligned to sound onset for both light and dark conditions (Figure 5C). In the light condition, an onset peak of 74.92 Hz occurred within 0–30 ms, followed by a sustained plateau averaging 11.33 Hz from 50 to 800 ms. The baseline firing rate was 2.71 Hz, and post-stimulus activity returned to 2.92 Hz. In contrast, the dark condition exhibited a markedly higher onset peak (122.37 Hz) and a more robust sustained response (18.74 Hz, 55–1000 ms), with baseline elevated to 4.20 Hz and post-stimulus return to 4.05 Hz. These results demonstrate that darkness potentiates both transient and sustained components of auditory-evoked activity in V2 neurons.

Figure 5D illustrates representative raster plots of spike discharges from a typical channel. Under both bright and dark conditions, the spike firing of V2 neurons appeared relatively uniform and stable, with more frequent firing observed in the dark environment. The addition of the auditory stimulus elicited noticeable burst firing patterns.

These results demonstrate that even under anesthesia, changes in environmental conditions can modulate the animal’s audiovisual perception. The significant increase in the firing rate of V2 neurons indicates that a greater number of neurons were recruited into an active state, and the concurrent waveform changes provide complementary evidence at the single-cell biophysical level that auditory stimuli engage active modulation of V2 membrane excitability and ion channel function.

### 3.4. Analysis of Distinctive Electrical Signals from Adjacent Recording Sites

**Figure 6:**
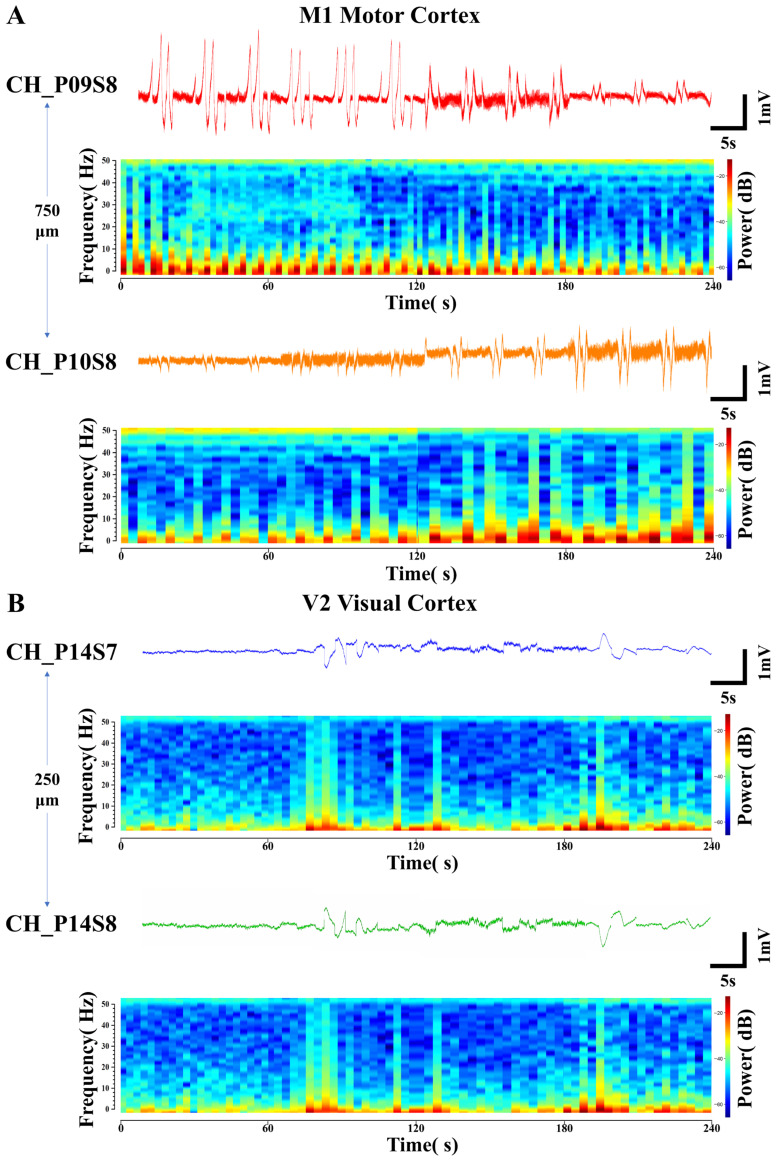
Specialized electrical signals from adjacent recording sites. (**A**) Opposing-trend local field potential signals and their corresponding time-frequency power spectrograms detected at sites with a lateral spacing of 750 μm in the M1 motor cortex. (**B**) Polarity-inverted local field potential signals and their corresponding time-frequency power spectrograms detected at sites with a longitudinal spacing of 250 μm in the V2 visual cortex.

During the processing of electrophysiological signals, two specialized phenomena were identified: opposing temporal trends in local field potential (LFP) amplitude between adjacent recording sites (Figure 6A) and polarity inversion of LFPs (Figure 6B). Power spectral analysis—a method that transforms signals into power spectra across frequencies to resolve LFPs in the frequency domain, reflecting energy density at different frequencies—was employed. Corresponding time-frequency power spectrograms (with a 50 Hz power line noise notch) were generated to illustrate the temporal variations in frequency and power spectral density (PSD) associated with these two phenomena.

In Figure 6A, the LFP signals were detected at site S8 on probes P09 and P10 located within the M1 motor cortex. The two sites were spaced 750 μm apart. Under the same four environmental stimulus conditions, this pair of LFP signals consistently exhibited opposing trends of gradually increasing and decreasing amplitude. Their corresponding PSDs also showed opposing trends in the intensity of high- and low-frequency electrical activity. This reflects differential activation patterns among distinct neuronal subpopulations within the regions covered by the electrode sites. CH_P09S8 might be situated closer to a hotspot of synaptic input from excitatory neuronal ensembles, whereas CH_P10S8 could be located within an area dominated by inhibitory interneurons. Such spatial functional differentiation results in the recording of opposing LFP trends from the two channels [51].

In Figure 6B, the LFP signals exhibiting polarity inversion were detected at the two sites (S7 and S8) closest to the surface of the V2 visual cortex region on the same probe P14, with an inter-site spacing of 250 μm. Their trends across the four environmental conditions are consistent with the patterns previously described, yet their amplitudes are completely opposite in sign. The PSDs corresponding to this pair of LFP signals are identical across different frequencies of electrical activity intensity. This indicates that the signals differ only in polarity. The electrical signals underlying neuronal information transfer involve current dipoles, with one side acting as a current sink (relatively negative potential) and the other as a current source (relatively positive potential). The postsynaptic potentials of neuronal populations establish a current source–sink distribution in the extracellular space. When functionally coupled ensembles of excitatory and inhibitory neurons are in close spatial proximity but generate currents in opposite directions, electrode sites positioned over their respective “source” and “sink” will record field potentials of opposite polarity [52]. The ability to capture such fine-grained activity patterns, which reflect the functional architecture of local microcircuits, further demonstrates the high-density recording capability of the flexible cortical electrode array.

## 4. Discussion

The 128-channel comb-shaped flexible cortical micro-electrode array developed in this study integrates a Parylene flexible substrate with a flexible thin-film microprobe array structure, enabling high-quality synchronous acquisition of electrocorticography (ECoG), intracortical local field potentials (LFPs), and neuronal action potentials (spikes). This design reduces mechanical tissue damage while balancing spatial resolution and modal completeness in signal acquisition, thereby partially bridging the technological gap between traditional flexible surface electrodes and rigid depth electrodes. Experimental results demonstrate that electrodes modified with platinum black nanoparticles exhibit significantly reduced impedance at 1 kHz. Furthermore, they stably record physiologically meaningful neural signals at various implantation depths, validating their utility in cross-modal sensory integration research.

Compared to existing technologies, this electrode achieves a favorable balance between flexibility, minimal invasiveness, and high channel density. Unlike rigid devices such as the Utah array, its fully flexible structure better conforms to the biomechanical properties of brain tissue, potentially mitigating chronic glial scar formation and signal attenuation associated with long-term implantation. This hypothesis, however, requires direct validation through future chronic histological studies. Conversely, compared to flexible ECoG electrodes limited to surface recording, its flexible thin-film microprobe structure provides essential shallow-penetration capability, allowing access to unit-level spiking activity closer to the neurons. Notably, the recording of opposing-trend and polarity-inverted local field potentials from adjacent channels suggests the array’s potential for resolving cortical micro-scale excitation–inhibition dynamics and current source–sink distributions. This offers a new tool for investigating neural microcircuit functions at the sub-millimeter level.

Despite these promising results, several limitations of the present study should be acknowledged. First, the evaluation was conducted primarily in acute anesthetized preparations. While anesthesia provides physiological stability, it may modulate baseline neural excitability and sensory processing networks, potentially influencing the observed responses to cross-modal stimuli. Future experiments in awake, behaving animals are necessary to confirm the array’s performance under more naturalistic conditions. Second, although stereotaxic techniques were employed, the manual surgical implantation process and the precise control of microprobe penetration depth inherently carry a degree of variability. The development of more standardized, potentially robotic-assisted implantation protocols could enhance reproducibility. Third, while the electrode demonstrated excellent electrophysiological performance acutely, its long-term biocompatibility, chronic recording stability, and the dynamic evolution of the tissue–electrode interface require comprehensive evaluation through extended implantation studies. Fourth, while the flexibility of the electrode was demonstrated through qualitative conformability tests, quantitative mechanical characterization—such as stress–strain curves and Young’s modulus measurements—was not performed. Incorporating such detailed mechanical evaluations in future work would provide a more rigorous basis for correlating device mechanics with tissue compliance. Fifth, this study did not assess the acute insertion footprint of the microneedle array, including immediate tissue displacement, vascular damage, or the ensuing acute inflammatory response. Evaluating these factors is essential for understanding the initial tissue reaction to the implant and for predicting long-term integration outcomes.

Future research should therefore focus on addressing these limitations through material–structure co-optimization, systematic chronic in vivo evaluations, and the development of less invasive implantation methodologies. Integration with wireless signal transmission systems will also be crucial for advancing the practical application of such flexible, minimally invasive hybrid electrodes in fields like closed-loop brain–computer interfaces and the long-term dynamic monitoring of neurological states.

## 5. Conclusions

In this study, a 128-channel flexible micro-electrode array with a comb-shaped structure was successfully designed and fabricated. Utilizing a Parylene flexible substrate with excellent biocompatibility, this device integrates a flexible thin-film microprobe array with surface electrodes. Unlike previous flexible probes that primarily focused on reducing mechanical mismatch, the unique contribution of this work lies in the architectural design of the monolithic array: by distributing both the probes and pure surface contact sites on the same Parylene substrate, this device achieves synchronized multi-scale signal recording of ECoG, LFP, and action potentials from the cortical surface and shallow layers. This integrated configuration allows the array to capture unit-level activity without relying on rigid shanks, while maintaining full structural compliance with brain tissue. This approach directly addresses the long-standing technical dilemma where traditional flexible surface electrodes are confined to field potentials and rigid implantable electrodes provoke progressive signal degradation due to tissue trauma.

Following modification with platinum black nanoparticles, the electrode interfacial impedance was significantly reduced from 157.03 kΩ to 3.64 kΩ at 1 kHz, accompanied by a marked improvement in the signal-to-noise ratio. This enhancement empowers the array to simultaneously capture high-quality, broad-spectrum neural electrical activity. Supported by its high-density layout, the array demonstrated excellent spatial resolving power for local field potentials. During experiments, it successfully recorded specialized signal features, including opposing temporal trends and polarity inversion between adjacent channels, confirming its utility for analyzing excitation–inhibition dynamics within cortical microcircuits. In audiovisual cross-modal stimulation experiments, the electrode exhibited stable signal acquisition performance and good environmental adaptability, validating its practical value for research into complex neural coding.

This research provides a novel device-based strategy to address the persistent conflict between “high signal quality and low tissue damage” in neural interfaces. It also establishes a significant methodological foundation for in-depth analysis of multi-level neural information processing mechanisms, the development of high-performance brain–computer interface systems, and advancements in clinical neural monitoring technologies. Future work may focus on further exploration in areas such as the long-term implantation biocompatibility of the device, integration with wireless signal transmission systems, and applications in closed-loop neuromodulation. Collectively, these advances—including the heterogeneous monolithic array presented herein—are converging toward next-generation neural interfaces that balance signal fidelity, spatial coverage, and long-term biocompatibility.

## Data Availability

Data are contained within the article.
